# Supplementary material for: The phased pan-genome of tetraploid European potato
Source: Nature. 2025 Apr 16;642(8067):389–97. doi: 10.1038/s41586-025-08843-0 (PMC12158759; doi:10.1038/s41586-025-08843-0)
Supplement: Supplementary file 5 — Supplementary Figs. 3–14, 20, 21, 23–27 and 34–44. [file 41586_2025_8843_MOESM5_ESM.zip › suppl_figure_25.pdf]

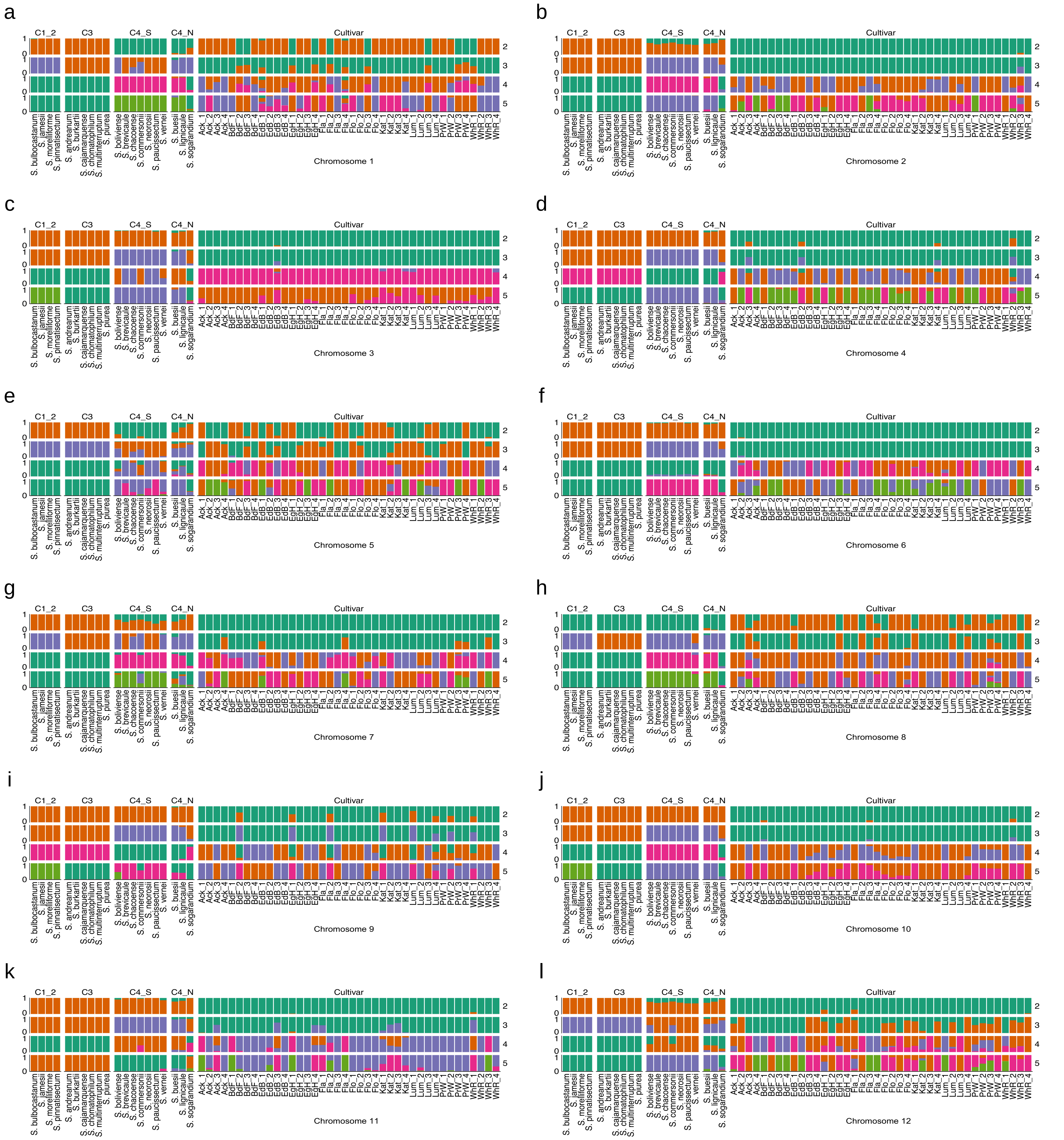

a
C1_2
C3
C4_S
C4_N
Cultivar
1
0
2
3
4
5
S. bulbocastanum
S. jamesii
S. morelliforme
S. pinnatisectum
S. andreanum
S. burkartii
S. cajamarquense
S. chomatophilum
S. multinterruptum
S. piurea
S. boliviense
S. brevicaule
S. chacoense
S. commersonii
S. neorosii
S. paucissectum
S. vernei
S. buesii
S. lignicaule
S. sogarandium
Ack_1
Ack_2
Ack_3
Ack_4
BdF_1
BdF_2
BdF_3
BdF_4
EdB_1
EdB_2
EdB_3
EdB_4
EgH_1
EgH_2
EgH_3
EgH_4
Fla_1
Fla_2
Fla_3
Fla_4
Flo_1
Flo_2
Flo_3
Flo_4
Kat_1
Kat_2
Kat_3
Kat_4
Lum_1
Lum_2
Lum_3
Lum_4
PrW_1
PrW_2
PrW_3
PrW_4
WhR_1
WhR_2
WhR_3
WhR_4
Chromosome 1
b
Chromosome 2
c
Chromosome 3
d
Chromosome 4
e
Chromosome 5
f
Chromosome 6
g
Chromosome 7
h
Chromosome 8
i
Chromosome 9
j
Chromosome 10
k
Chromosome 11
l
Chromosome 12
